# Recalibrating disease parameters for increasing realism in modeling epidemics in closed settings

**DOI:** 10.1186/s12879-016-2003-3

**Published:** 2016-11-14

**Authors:** Livio Bioglio, Mathieu Génois, Christian L. Vestergaard, Chiara Poletto, Alain Barrat, Vittoria Colizza

**Affiliations:** 1 Santé Publique France, French National Public Health Agency, Saint-Maurice, France; 2 Aix Marseille Univ, Université Toulon, CNRS, CPT, Marseille, France; 3 Sorbonne Universités, UPMC Univ Paris 06, INSERM, Institut Pierre Louis d’Epidémiologie et de Santé Publique (IPLESP), Paris, France; 4 ISI Foundation, Turin, Italy

**Keywords:** Modeling, Infectious diseases, Homogeneous mixing, Contact network

## Abstract

**Background:**

The homogeneous mixing assumption is widely adopted in epidemic modelling for its parsimony and represents the building block of more complex approaches, including very detailed agent-based models. The latter assume homogeneous mixing within schools, workplaces and households, mostly for the lack of detailed information on human contact behaviour within these settings. The recent data availability on high-resolution face-to-face interactions makes it now possible to assess the goodness of this simplified scheme in reproducing relevant aspects of the infection dynamics.

**Methods:**

We consider empirical contact networks gathered in different contexts, as well as synthetic data obtained through realistic models of contacts in structured populations. We perform stochastic spreading simulations on these contact networks and in populations of the same size under a homogeneous mixing hypothesis. We adjust the epidemiological parameters of the latter in order to fit the prevalence curve of the contact epidemic model. We quantify the agreement by comparing epidemic peak times, peak values, and epidemic sizes.

**Results:**

Good approximations of the peak times and peak values are obtained with the homogeneous mixing approach, with a median relative difference smaller than 20 *%* in all cases investigated. Accuracy in reproducing the peak time depends on the setting under study, while for the peak value it is independent of the setting. Recalibration is found to be linear in the epidemic parameters used in the contact data simulations, showing changes across empirical settings but robustness across groups and population sizes.

**Conclusions:**

An adequate rescaling of the epidemiological parameters can yield a good agreement between the epidemic curves obtained with a real contact network and a homogeneous mixing approach in a population of the same size. The use of such recalibrated homogeneous mixing approximations would enhance the accuracy and realism of agent-based simulations and limit the intrinsic biases of the homogeneous mixing.

**Electronic supplementary material:**

The online version of this article (doi:10.1186/s12879-016-2003-3) contains supplementary material, which is available to authorized users.

## Background

Mathematical models provide a theoretical framework that can be applied to improve our understanding of the spread of infectious diseases in a host population [1–4]. A vast range of approaches has recently been developed for the analysis and interpretation of epidemic data, characterization of transmission dynamics, contingency planning, evaluation of intervention strategies and support of disease outbreak management [3, 4].

Different degrees of resolution are considered in these methods, ranging from simple homogeneous mixing models to data-demanding high-resolution approaches [3]. Among the latter, agent-based models [5] push the modeling strategy to numerically recreating synthetic populations with high accuracy at the individual level [6–16]. They yield detailed predictions of the spatial spread of the epidemic and of the effectiveness of a variety of intervention strategies, aiming for higher realism. They describe the biological, social and behavioral aspects of the epidemic process explicitly including individual features, based on available knowledge (e.g. surveys, statistics) and assumptions where data is missing. Individuals are followed in time during their daily activities and their infection status is updated depending on the contacts they establish, along which transmission may occur.

Contacts take place within different mixing groups and with associated mixing rates, defined on available data and depending on the considered method. Most agent-based approaches define four types of mixing groups – namely, *home*, *school*, *workplace*, and *community* (see e.g. [10]). The first three determine the contacts occurring among individuals in each of those locations. *Community* accounts for all other contacts that individuals may casually establish during the day. Mixing groups are built based on available statistics profiling the population under study and providing the frequency, type and location of households, schools, and workplaces of various sizes [10]. Homogenous mixing is then generally assumed in each group, given the lack of explicit contact data for all these settings at a country scale.

Several theoretical and data-driven studies have however highlighted the limitation of homogeneous mixing assumptions in many instances. It was shown to be unrealistically simple, lacking the description of any sort of individual heterogeneity (e.g., number of contacts, but also frequency, duration, timeline of contacts, etc.) [17–27]. Other works have focused on the impact that homogeneous mixing approximations may have in the resulting epidemic dynamics compared to more realistic settings (e.g. heterogeneity in contacts, explicit contact patterns, and others). Aiming to improve the accuracy of the approximation, they proposed possible modifications of the mean-field equations to effectively account for the network structure of contacts in the population or the direct comparison of different network structures [17]. For instance, they incorporated the basic reproductive number as a parameter in the equations [28], used time-dependent transmission rates [29] or non-linear modifications of the infection term accounting for heterogeneity [30–32]. While some of these approximations performed well given sufficient epidemiological data, they were found to be generally not accurate enough to capture the disease dynamics across a wide range of population contact patterns and disease transmission rates [17]. Most importantly, these approaches were tested on static synthetic contact networks, and the case of time-resolved empirical or synthetic contact data has not been addressed so far. As a growing body of literature has shown, epidemic processes on time-varying networks present however a number of specificities and differences compared with spreading processes on static networks [20, 33–42]. Understanding whether homogeneous mixing approximations can reproduce epidemics on temporal networks has thus a clear interest to increase the realism of these approximations and of larger-scale approaches adopting them, such as e.g. agent-based models.

Though high-resolution time-resolved contact data are still too rare to comprehensively feed agent-based simulations at large spatial scales, a vast research effort has indeed recently allowed mining such data in a variety of closed settings, using wearable sensors and digital devices [33, 43–46]. Monitored settings include, among others, schools [26, 33, 47–49], workplaces [50], hospitals [24, 25, 46], conferences [20, 51], and museums [51, 52]. Such data thus offer a yet unexploited opportunity to compare, with respect to the course of an epidemic, the homogeneous assumption generally used in mixing groups to realistic time-resolved contact patterns tracked in specific settings. Taking this further, they allow us to explore whether it is possible to adjust the disease parameter values of a compartmental model under a homogeneous mixing assumption in order to reproduce the epidemic simulated on the contact data. For example, how can we best replicate an epidemic unfolding on empirical time-resolved contacts among *N* students at school by simply using a homogeneous assumption for a mixing group of *N* individuals with tuned parameters? Answering this question would provide an alternative and simpler description of the complex pattern of interactions for epidemic purposes, for the specific setting under study.

Here we focus on empirical data collected in a variety of settings. To be able to generalize our results to categories of settings with different characteristics (e.g. different population sizes), we test our approach on datasets of different sizes and group structures, investigating how the fitted parameter values of the homogeneous mixing model depend on the sizes of groups, the number of groups, and on the disease parameters. We consider contact data from a school, a workplace, and a scientific conference. Furthermore, a generative model of synthetic time-resolved contacts is used to validate the findings and to explore variations in population size and group structures beyond what is allowed by the empirical datasets.

The aim of our work is twofold: *(i)* to deepen our understanding of the limitations of homogeneous approximations in real situations; and *(ii)* to offer a tool to systematically improve the realism and accuracy of epidemic simulations that can be used in modeling approaches where closed settings are explicitly considered, as for instance in spatial agent-based models.

## Methods

### Contact networks

Here we present the empirical contact datasets and the synthetic contact model that will be used to construct temporal contact networks for the epidemic spreading simulations.

#### Empirical contact data

Empirical contact data have been collected by the SocioPatterns collaboration [53] making use of wearable sensors embedded in badges that exchange ultra-low power radio packets for detecting face-to-face proximity between individuals [44]. Here we consider three datasets among those collected by SocioPatterns, each representing a different social setting: a workplace, a school and a scientific conference. The workplace data were collected in an office building in France during two weeks in 2013 (from June 24 to July 5, 2013). The population under study was composed of individuals from 5 departments of 15,26,34,13 [50, 54]. The school data were collected in a French high school, where 9 classes of similar sizes were tracked during the week of Dec. 2 to 6, 2014 [49, 55]. The conference data were collected during the 2009 Annual French Conference on Nosocomial Infections on June 3, 2009 [20]. The school and workplace datasets have a community structure [49, 50], while the conference does not [20]. Contact data recorded with a temporal resolution of 20 seconds are represented by temporal contact networks: each individual is represented as a node, and a link is drawn between two nodes at time *t* if a contact has been recorded between them at that time. Here we keep the highest temporal resolution given by the data. Temporal aggregation has already been studied in the realm of epidemic spreading and other dynamical processes, and it was shown to alter the process outcome under certain conditions [20, 42, 56]. To focus exclusively on the aspect of recalibration and be able to span a large range of values of the timescale of the epidemic dynamics, we choose here to use the full time-resolved dataset, as also done in previous works [20, 33, 57, 58], in order to avoid any such effect that could potentially impact our results.

To study the effect of population size, we also consider subsets of each full dataset. Specifically, we consider: two subsets composed of 4 and 3 departments for the workplace; two subsets composed of 6 and 3 classes for the school; two subsets composed of 75 % and of 50 % of the population tracked at the conference (randomly chosen individuals). In the workplace and conference cases, subsets are not mutually exclusive. The subsets are extracted from the full datasets by considering only the contacts occurring among the selected individuals. Sizes of each dataset and of the associated subsets are summarized in Table 1.

**Table 1:** Details of empirical contact data at different locations

Location	Set	Size *N*	#Groups	Group sizes	Duration
Workplace	Full	92	5	15, 34, 4, 26, 13	2 weeks
	Subset 1	79	4	15, 34, 4, 26	
	Subset 2	51	3	34, 4, 13	
School	Full	326	9	37, 33, 39, 33, 29, 38	1 week
				44, 39, 34	
	Subset 1	212	6	33, 39, 29, 38, 39, 34	
	Subset 2	114	3	37, 33, 44	
Conference	Full	403	1	403	1 day
	Subset 1	303	1	303	
	Subset 2	202	1	202	

#### Synthetic contact model

To construct synthetic temporal contact networks, we extend the agent-based model of Vestergaard et al. [59] to model a population divided into social groups, with contacts occurring preferentially between individuals of the same group. The model generates temporal contact networks that are similar to empirical ones. Namely, it produces heterogeneous contact and inter-contact durations and heterogeneous frequencies of contacts between pairs of individuals, as observed in many realistic situations [59].

Our model considers a population of *N* agents divided into *Q* non-overlapping groups. We denote by *n*_*q*_ the number of agents in group $q~\left (\sum _{q=1}^{Q}n_{q}=N\right)$. If two nodes *i* and *j* are in contact, the link (*i,j*) is *active*, otherwise it is *inactive*. We denote by *t*_(*i,j*)_ the last time the link (*i,j*) changed its state (from active to inactive or vice versa) and by *t*_*i*_ the last time when a link from/to agent *i* changed its state. Agents at time *t* are characterized by the time *τ*_*i*_=*t*−*t*_*i*_ elapsed since the last time the agent either gained or lost a contact. Links are characterized by the time *τ*_(*i,j*)_=*t*−*t*_(*i,j*)_ elapsed since the link was last either activated or inactivated.

We initialize the network with all agents isolated (i.e. all links inactive). We set *t*_*i*_=0 and *t*_(*i,j*)_=0 for all agents and links, respectively. At each time step *Δ* *t*, all active links and all agents are updated as follows:

(i) Each active link (*i,j*) is inactivated with probability *Δ* *t* *z* (1+*τ*_(*i,j*)_)^−1^, where the parameter *z* controls the rate with which contacts end;

(ii) Each agent *i* initiates a contact with another agent with probability *Δ* *t* *b* (1+*τ*_*i*_)^−1^. The other agent *j* is chosen among agents that are not in contact with *i*, with probability proportional to *a*_*p,q*_(1+*τ*_*j*_)^−1^(1+*τ*_(*i,j*)_)^−1^. Here *b* controls the rate of contact creation, while *p* and *q* are the groups which *i* and *j* belong to, respectively, and *a*_*p,q*_ regulates the probability for an agent of group *p* to create a contact with an agent of group *q*. Since the contact networks are undirected, we consider symmetrical values for these probabilities, i.e. *a*_*p,q*_=*a*_*q,p*_.

The model is used for generating temporal contact networks for nine synthetic populations, made by 3,6 and 9 groups of 10,20 and 30 individuals each (Table 2). In this way, we can separately study the influence of the total population size, the number of groups, and the number of individuals per group, as these parameters are more easily tunable than in empirical data. Population sizes range from 30 to 270 individuals, and we obtain three pairs of datasets with the same population size but different group compositions (e.g., datasets with total size equal to 90, obtained as 9 groups of 10 agents or 3 groups of 30 agents). For definiteness, we consider only two values of *a*_*p,q*_:*a*_*p,p*_=*a*_0_ for agents in the same group, and *a*_*p*≠*q*_=*a*_1_ for agents of different groups, and we consider groups of the same size, to provide a benchmark against which to compare the results of empirical data, where the group structure can be either homogeneous or not. The values of *a*_0_ and *a*_1_ were chosen such that agents had approximately 50 times as many contacts with agents from their own group as with agents from each of the other groups, comparably to the school setting.

**Table 2:** Details of synthetic contact data

Groups	Group size	Total size *N*
3	10	30
	20	60^+^
	30	90^∗^
6	10	60^+^
	20	120
	30	180^−^
9	10	90^∗^
	20	180^−^
	30	270

Symbols identify the pairs of groups with the same total population size

### Simulations of epidemic spread

We consider a standard Susceptible-Infectious-Recovered (SIR) epidemic model [1, 2], where individuals belong to one of the following compartments at any time step: susceptible (S), infectious (I) or recovered (R). A susceptible (S) individual in contact with an infectious (I) individual becomes infectious (I) at a given constant rate (called transmissibility), while an infectious (I) individual spontaneously recovers from infection with a constant rate (the recovery rate), entering the recovered (R) compartment.

#### Epidemics on contact networks

In this framework, the epidemic spread takes place on the temporal contact network (obtained from either empirical or synthetic contact data) with temporal resolution *τ*=20 s. Nights and weekends have been removed, in order to focus exclusively on the contact dynamics of interest and for which data are available. We denote by *β*_c_ the transmission rate of the infection from an infectious individual to a susceptible individual upon contact, and by *μ*_c_ the recovery rate of infectious individuals. Each simulation is stochastic and starts with one infected node (the seed) chosen randomly in a fully susceptible population. The seeding occurs at a randomly chosen time of the dataset.

A key metric to describe the epidemic is the basic reproductive number *R*_0_, defined as the number of secondary cases an average infectious individual generates over the course of its infectious period in a fully susceptible population [1, 2]. Here we compute the basic reproductive number $R_{0}^{\mathrm {c}}$ for epidemics evolving on contact networks as the number of susceptible individuals infected by the seed during its infectious period, averaged over all possible seeds. We explored the parameter space given by the reproductive number ${R_{0}^{\mathrm {c}}}$ and the recovery rate *μ*_c_. In practice, for each pair of desired values $(R_{0}^{\mathrm {c}},{\mu _{\mathrm {c}}})$, we first perform simulations at various *β*_c_ and fix the value of *β*_*c*_ such that the average number of secondary infections generated by the seed before recovering is equal to ${R_{0}^{\mathrm {c}}}$.

We record the fraction of infected individuals (epidemic prevalence) in the population at each time step for each realization, and consider the temporal evolution of its average (if needed, the dataset is repeated until the epidemic ends). We use the temporal Gillespie algorithm of [60] for numerical simulations, avoiding in this way the slowing down of usual algorithms when high-resolution temporal data are used in simulations with realistic epidemic parameters.

#### Homogeneous mixing model

Here we consider a frequency dependent homogeneous mixing compartmental model, based on a mass-action law for the transmission of the infection between individuals who are assumed to mix uniformly and randomly [1, 2]. No explicit pattern of contacts is considered. If we denote by *β* and *μ* the transmission and recovery rates, respectively, in this epidemic framework the state of each node evolves according to the two following transitions:

$$ S \xrightarrow{\beta \cdot \frac{I}{N}} I, \quad \quad I \xrightarrow{\mu }R.  $$

That is, at each infinitesimal time step *dt*, each susceptible individual becomes infectious with probability $\beta \cdot \frac {I(t)}{N}\,dt$, where *I*(*t*) is the number of infectious individuals in the population at time *t*, and each infectious individual recovers with probability *μ* *dt*. With respect to the epidemic simulated on contact networks described previously, here the rate of transmission *β* integrates implicitly both the average number of contacts per individual and the transmissibility per contact. The reproductive number is here simply given by ${R_{0}^{h}}=\beta /\mu $.

The infection dynamics is simulated using the classic Gillespie algorithm of [61], starting with one infected individual in a fully susceptible population of size *N*.

#### Fitting procedure

**Fig. 1:**
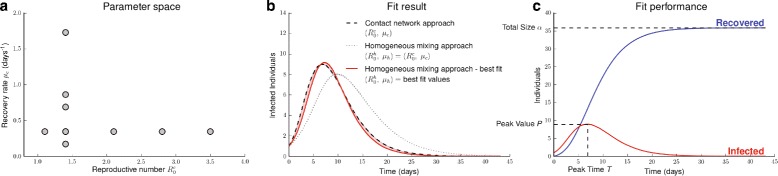
Fitting procedure. **a** Nine pairs of parameter values $({R_{0}^{\mathrm {c}}}, {\mu _{\mathrm {c}}})$ used in the simulations of epidemics on the temporal contact network. **b** Schematic visualization of the fitting procedure with results. We consider the average prevalence profile of an epidemic with parameters $({R_{0}^{\mathrm {c}}}, {\mu _{\mathrm {c}}})$ unfolding on the contact network (*black dashed line*). The homogeneous mixing approach with the same parameter values generally leads to a different average prevalence curve (*black dotted line*). By exploring the parameter space in the homogeneous mixing approximation, we find the values of $(R_{0}^{\mathrm {h}},\mu _{h})$ that best reproduce the average prevalence curve obtained on the contact network (*red continuous line*). **c** Graphical visualization of the three epidemic features used for the evaluation of the fit: peak time *T*, peak value *P* and total size *α*

Given an average prevalence curve obtained by simulating an epidemic on the contact network with parameters $(R_{0}^{\mathrm {c}}, \mu _{\mathrm {c}})$, we explore the parameter space $(R_{0}^{\mathrm {h}},\mu _{\mathrm {h}})$ of the homogeneous mixing model in order to find the pair that best reproduces the prevalence curve obtained with the contact network approach (Fig. 1).

We consider ranges of parameters typical of respiratory infections such as influenza [62] or Severe Acute Respiratory Syndrome (SARS) [63, 64], with ${R_{0}^{\mathrm {c}}}$ taking values between 1 and 4, and *μ*_c_ between 0.1 and 2 d^−1^. More in detail, we consider the following points in the parameter space $({R_{0}^{\mathrm {c}}}, {\mu _{\mathrm {c}}})$: ${R_{0}^{\mathrm {c}}}=1.1,\,1.4,\,2.1,\,2.8,\,3.5$ and *μ*_c_=0.3456d^−1^; ${R_{0}^{\mathrm {c}}}=1.4$ and *μ*_c_=0.1728, 0.3456,0.6912, 0.8640, 1.728d^−1^, for a total of nine points (Fig. 1 a).

For each point $({R_{0}^{\mathrm {c}}}, {\mu _{\mathrm {c}}})$, we simulate 10,000 SIR stochastic epidemics on the temporal contact network and compute the resulting average prevalence 〈*I*_*c*_(*t*)/*N*〉. We then explore the parameter space (*R*_0_,*μ*) and for each point we simulate 10,000 SIR stochastic epidemics in the homogeneous mixing approximation in a population having the same size *N*. We fit the obtained average prevalence 〈*I*_*h*_(*t*)/*N*〉 to 〈*I*_*c*_(*t*)/*N*〉 by minimizing the cumulative squared difference between the two curves using the Levenberg-Marquardt algorithm [65, 66]. Each iteration step of the fitting procedure corresponds to performing 10,000 stochastic realizations of the homogeneous mixing model with the estimate vector as input value. The estimate vector is composed of the fitted parameters of the epidemic $({R_{0}^{\mathrm {h}}},{\mu _{\mathrm {h}}})$, which are the values of the basic reproductive number *R*_0_ and recovery rate *μ* of the homogeneous mixing model that lead to the best fit of the average prevalence curve for the epidemic taking place on the contact network (Fig. 1 b).

To assess the results of the fit in a quantitative way that is directly meaningful in terms of epidemic risk and outcome of the disease spread, we inspect three features of the epidemic – namely the peak time *T* of the prevalence curve, the peak value *P*, and the total size *α* of the epidemic – and evaluate how they change between the two epidemic frameworks (see Fig. 1 c). As we will consider parameter values giving rise to epidemic sizes that span up to two orders of magnitude, we consider below the logarithm of *α*. The peak time and peak value are important to give a measure of the impact over time of an epidemic, whereas the epidemic size quantifies the epidemic’s overall impact in a population. For each feature *f*, we compute the relative difference *Δ*_*f*_ between the value obtained in the homogeneous mixing approximation and the one obtained in the contact epidemic model, used as a benchmark, i.e., $\Delta _{f}=\frac {f_{h}-f_{c}}{f_{c}}$. In the case of the epidemic size, our criterion for the evaluation of the fit performance is the same as the *epidemiological distance* defined in [17]. The fitting procedure is performed for epidemic simulations on each empirical dataset described in Table 1 and on each synthetic contact network described in Table 2.

## Results

We first examine the performance of the fit in terms of the three epidemic features (*T*, *P*, *α*) for each contact network considered. We will then evaluate the relation between the parameters in the homogeneous mixing framework resulting from the fit and the parameters used in the simulations on the contact network data, and assess how this recalibration depends on the social setting, the size of the population and the group structure.

### Performance of the fit

**Fig. 2:**
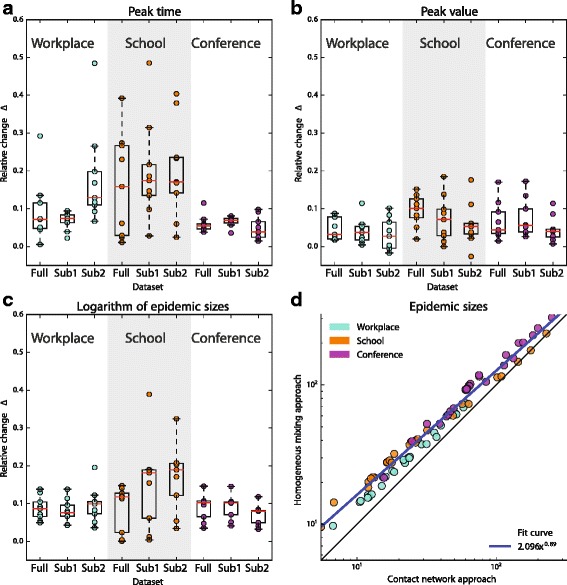
Performance of the fit for empirical data. **a**-**b**-**c** Variations of the three epidemic features (**a** *Δ*_*T*_, **b** *Δ*_*P*_, **c** *Δ*_log*α*_) for all empirical datasets under study. Each point corresponds to one set of parameters $({R_{0}^{\mathrm {c}}}, {\mu _{\mathrm {c}}})$. **d** Epidemic size obtained from the homogeneous mixing approach with best fit values as a function of the epidemic size resulting from the contact network epidemic simulations. Each point corresponds to one parameter set $({R_{0}^{\mathrm {c}}}, {\mu _{\mathrm {c}}})$ and one dataset. The *black line* corresponds to the diagonal, and the *blue line* is a fit to all the points (using unweighted least squares fitting of the logarithmic values)

For the empirical contact data, we find different behaviors of the relative difference *Δ*_*T*_ in the peak times depending on the dataset under study (Fig. 2 a). *Δ*_*T*_ is low in the conference setting (median <10 *%*) and displays low variability. In the school datasets we find larger median values (around 0.170) and larger dispersions, whereas in the workplace setting we obtain a mixed behavior, with small values and small variability in subset 1, and larger medians and variations in subset 2.

Less variation across the datasets is observed in the relative differences *Δ*_*P*_ of the peak values, with median values in the interval [0.027,0.101], for all datasets and subsets considered (Fig. 2 b).

Similarly to the peak time results, the largest variation in the epidemic sizes (*Δ*_log*α*_) is observed in the school setting, specifically in the two subsets, whereas the workplace and the conference display smaller median values. The largest relative differences correspond to small epidemic sizes, and a power-law relation can be found linking the epidemic size in the fitted homogeneous mixing model to the one resulting from the spread on the contact network, i.e., $\alpha _{h}=a{\cdot \alpha _{c}^{b}}$, with *a*=2.1±0.2 and *b*=0.89±0.02 (Fig. 2 d).

**Fig. 3:**
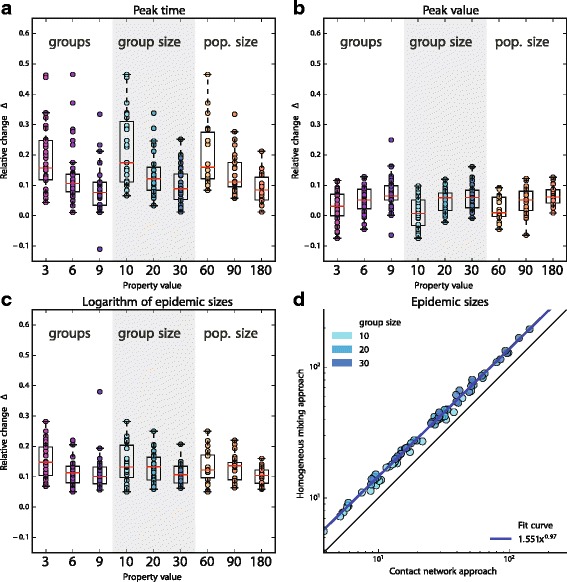
Performance of the fit in synthetic populations. **a**-**b**-**c** Variations of the three epidemic features (**a** *Δ*_*T*_, **b** *Δ*_*P*_, **c** *Δ*_log*α*_) for all synthetic datasets under study. Each point corresponds to one set of parameters $({R_{0}^{\mathrm {c}}}, {\mu _{\mathrm {c}}})$. Numbers on the x-axis give the value of the relevant property of the data (from left to right, number of groups, number of individuals per group or total population size), see Table 2. **d** Epidemic size obtained from the homogeneous mixing approach with best fit values as a function of the epidemic size resulting from the contact network epidemic simulations. Each point corresponds to one parameter set $({R_{0}^{\mathrm {c}}}, {\mu _{\mathrm {c}}})$ and one dataset. The *black line* corresponds to the diagonal, and the *blue line* is a fit to all the points (using unweighted least squares fitting of the logarithmic values)

When the fit is performed on the epidemics occurring on the synthetic contact networks, we observe a stable behavior across the various epidemic features and datasets explored (Fig. 3). Here we investigate the effect of varying the numbers of groups, the group sizes and the total population sizes (see Table 2). The approximations of the peak time obtained with the homogeneous mixing approach show a median relative difference *Δ*_*T*_ ranging between 10 *%* and 17.5 *%*, and a decrease of both the median and the variability of *Δ*_*T*_ as the number of groups, the group size or the total population increase (Fig. 3 a). The approximations of the peak value show even smaller relative variations, with all medians of *Δ*_*P*_ below 10 % and small dispersion, even if *Δ*_*P*_ increases slightly with the dataset size (Fig. 3 b). Results for the epidemic size are qualitatively similar to the ones obtained for the peak time. The median value of *Δ*_log*α*_ generally decreases when the number of groups, group size or total population size increases, and in all cases remains below 15 %. The relation between *α*_*c*_ and *α*_*h*_ is close to linear ($\alpha _{h}=a\cdot {\alpha _{c}^{b}}$, with *a*=1.55±0.05 and *b*=0.975±0.006, Fig. 3 d), where the fit is performed considering all the datasets listed in Table 2 and parameter values of Fig. 1 a.

We tested for correlations between the three indicators of the performance of the fit (Table 3 and Figure S1 of Additional file 1). Significant negative correlations were found between *Δ*_*P*_ and *Δ*_*T*_ for the School dataset (Pearson correlation coefficient and 95 % confidence interval *r*=−0.9[−1.0,−0.5], *p*=0.003), and between *Δ*_*P*_ and *Δ*_log*α*_ for the Conference dataset (*r*=−0.9[−1.0,−0.7], *p*<10^−3^). Positive correlations were found between *Δ*_log*α*_ and *Δ*_*T*_ for the Workplace (*r*=0.8[0.4,1.0], *p*=0.004) and School (*r*=0.8[0.2,1.0], *p*=0.02). All other cases were qualitatively similar but non-significant.

**Table 3:** Correlations between relative differences

Location (Full)	*Δ*_*P*_ vs. *Δ*_*T*_		*Δ*_log*α*_ vs. *Δ*_*T*_		*Δ*_*P*_ vs. *Δ*_log*α*_	
	*r*[95 *%* *CI*]	*p*-value	*r*[95 *%* *CI*]	*p*-value	*r*[95 *%* *CI*]	*p*-value
Workplace	−0.2[−0.8,0.5]	0.6	0.8[0.4,1.0]	0.004	−0.02[−0.68,0.65]	0.95
School	−0.9[−1.0,−0.5]	0.003	0.8[0.2,1.0]	0.02	−0.5[−0.9,0.2]	0.2
Conference	−0.2[−0.8,0.5]	0.6	0.3[−0.4,0.8]	0.4	−0.9[−1.0,−0.7]	<10^−3^

Finally, we explored the dependence of the fit performance on the epidemic parameters. We found that for all three datasets *Δ*_log*α*_ is negatively correlated with ${R_{0}^{c}}$ (Tables S1 and S2, and Figure S2 of Additional file 1). Additional significant results emerged in the School dataset, displaying a negative correlation between *Δ*_*T*_ and the reproductive number, and in the Conference dataset, yielding a positive correlation between *Δ*_*P*_ and ${R_{0}^{c}}$. Non-significant associations were found between the variations and the recovery rate *μ*_*c*_.

### Rescaling the parameters of the homogeneous mixing model

**Fig. 4:**
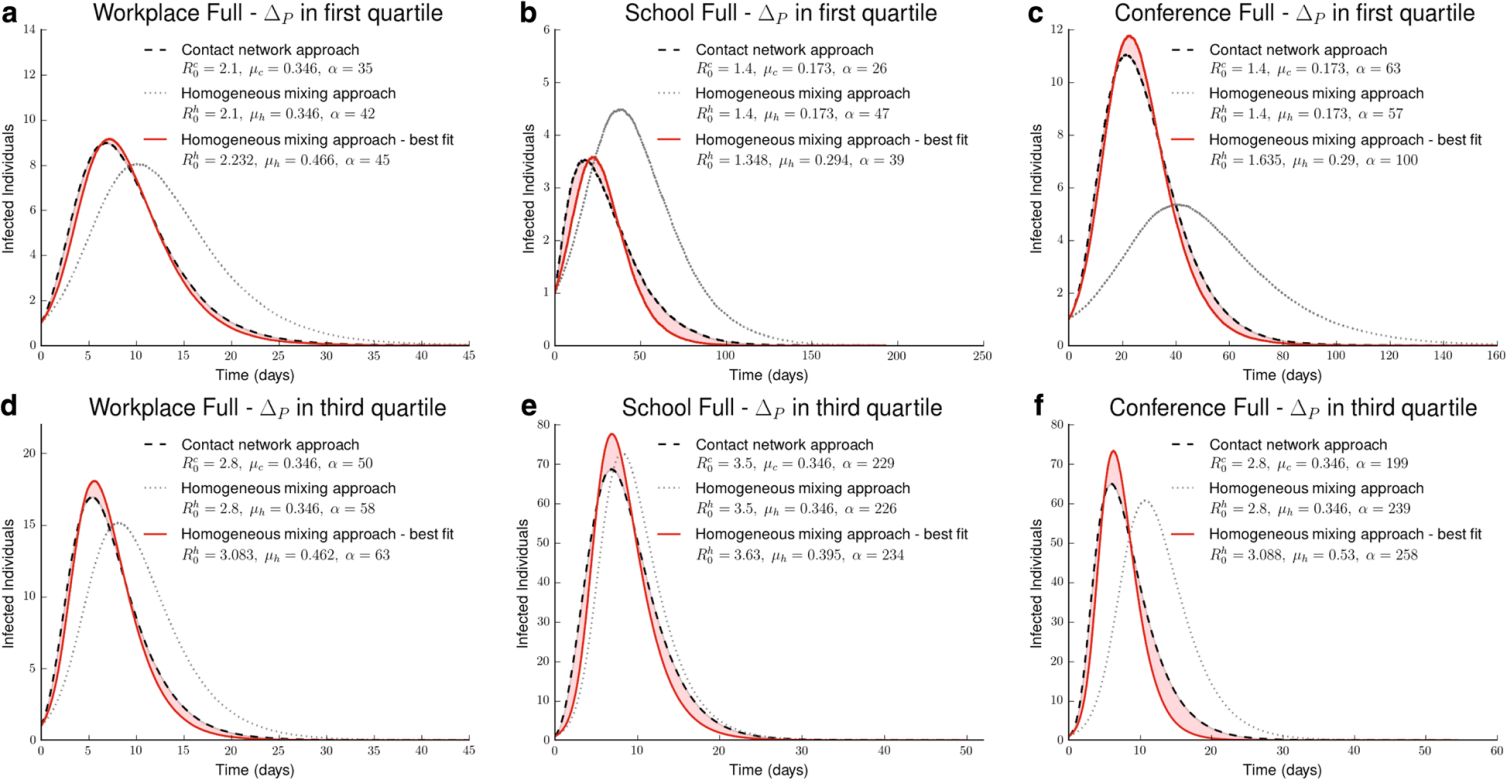
Examples of fit results. Fitting procedure in Workplace full dataset, **a** and **d**, School full dataset, **b** and **e**, and Conference full dataset, **c** and **f**. For each location we represent a sample of a “good” fit (**a**, **b** and **c**) and a sample of a “bad” fit (**d**, **e** and **f**), i.e. fits yielding a relative difference of peak value *Δ*_*P*_ in the first and in the third quartile of Fig. 2, respectively. For each case, the average prevalence curve is shown, with the values of the parameters *R*_0_ and *μ* and the average epidemic size *α*

After having quantified the accuracy of the homogeneous mixing approach in reproducing an epidemic taking place on a contact network, we focus here on the fit results. Figure 4 shows six examples of prevalence profiles obtained from the fit procedure with the full empirical datasets, for cases resulting in either large or small variations of the peak value (*Δ*_*P*_ in the first or third quartile of its distribution, respectively). We find that the outcome of the epidemic described by the homogeneous mixing that best captures the contact network epidemic is generally strongly different from the one obtained with a simple homogeneous model with unchanged parameter values (i.e. using $({R_{0}^{\mathrm {h}}},{\mu _{\mathrm {h}}})=({R_{0}^{\mathrm {c}}},{\mu _{\mathrm {c}}})$). The initial rise of the epidemic is found to be faster in the epidemic on contact networks (and thus in the associated best fit homogeneous mixing) with respect to the unaltered homogeneous approximation. The recalibration of the parameters needed to fit the curve of the epidemic on the contact network does however not act only on the reproductive number: a tuning of the recovery rate is also needed to capture the whole timeline of the original epidemic dynamics. Accounting for such changes leads to strong differences in the duration of the epidemic of the recalibrated vs. unaltered homogeneous mixing approach in some of the cases explored (see e.g. panels (b) and (c)).

Given the changes in the spreading parameters required to capture the epidemic dynamics evolving on the time-resolved network of contacts by a homogeneous mixing framework, in the following we systematically explore how the fitted values of the epidemic parameters $({R_{0}^{\mathrm {h}}},{\mu _{\mathrm {h}}})$ depend on the values $({R_{0}^{\mathrm {c}}},{\mu _{\mathrm {c}}})$ of the original spreading simulations.

**Fig. 5:**
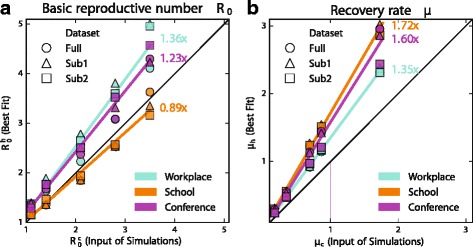
Recalibration of epidemic parameters for empirical networks. **a** Best fit values of the reproductive number ${R_{0}^{\mathrm {h}}}$ as functions of the corresponding input values ${R_{0}^{\mathrm {c}}}$. **b** Best fit values of the recovery rate *μ*_*h*_ as functions of the corresponding input values *μ*_*c*_. *Symbol shapes* correspond to datasets. For ${R_{0}^{\mathrm {c}}}=1.4$ in **a**, we show the value of ${R_{0}^{\mathrm {h}}}$ averaged over the various values of *μ*_*c*_ considered; for *μ*_c_=0.346 in **b**, we show the value of *μ*_h_ averaged over the various values of ${R_{0}^{\mathrm {c}}}$ considered. Lines are the result of a linear fit to all points obtained in a given location using unweighted least squares

We show in Fig. 5 a the fitted values of ${R_{0}^{\mathrm {h}}}$ for fixed *μ*_c_=0.3456d^−1^ and for varying $R_{0}^{\mathrm {c}}=1.1, 1.4, 2.1, 2.8, 3.5$ (see Fig. 1 a). Conversely, in Fig. 5 b we show the values of *μ*_h_ obtained at fixed ${R_{0}^{\mathrm {c}}}=1.4$ and varying *μ*_*c*_. Note that *μ*_h_ is approximately constant when *μ*_c_ is kept fixed and ${R_{0}^{\mathrm {c}}}$ is varied, while ${R_{0}^{\mathrm {h}}}$ is approximately constant when $R_{0}^{\mathrm {c}}$ is fixed and *μ*_c_ is varied (not shown).

The results uncover the presence of a linear dependency existing between the parameters of the homogeneous mixing model and the input parameters of the contact network approach, for all cases under study. The relation between the reproductive numbers ${R_{0}^{\mathrm {c}}}$ and ${R_{0}^{\mathrm {h}}}$ at fixed *μ*_c_ (Table 4) shows that in order to reproduce the prevalence curve, the homogeneous mixing model requires an increase of the transmissibility of the epidemic in the conference and workplace settings, and instead a slight decrease in the case of the school. We note moreover that the required increase is similar in the workplace and conference contexts (workplace: 1.36±0.07; conference: 1.23±0.05). The linear relations found between the recovery rates *μ*_c_ and *μ*_h_ are based on scaling factors all larger than one (ranging from 1.346 to 1.72, see Table 4), indicating that larger values for this parameter are always required by the homogeneous mixing approximation. Different subsets of a given location yield slightly different slopes compared to the corresponding full dataset (not shown).

**Table 4:** Values and standard deviations of the parameters *a*, *b*, *d*, *e* of the linear relations ${R_{0}^{\mathrm {h}}}=a\cdot {R_{0}^{\mathrm {c}}} + b$ and *μ*_h_=*d*·*μ*_c_+*e* obtained from the fits of Fig. 5

Location	*a*	*b*	*d*	*e*
Workplace	1.36±0.07	−0.2±0.1	1.346±0.005	0.007±0.005
School	0.89±0.07	0.1±0.1	1.72±0.04	0±0.04
Conference	1.23±0.05	0±0.1	1.60±0.01	−0.020±0.009

**Fig. 6:**
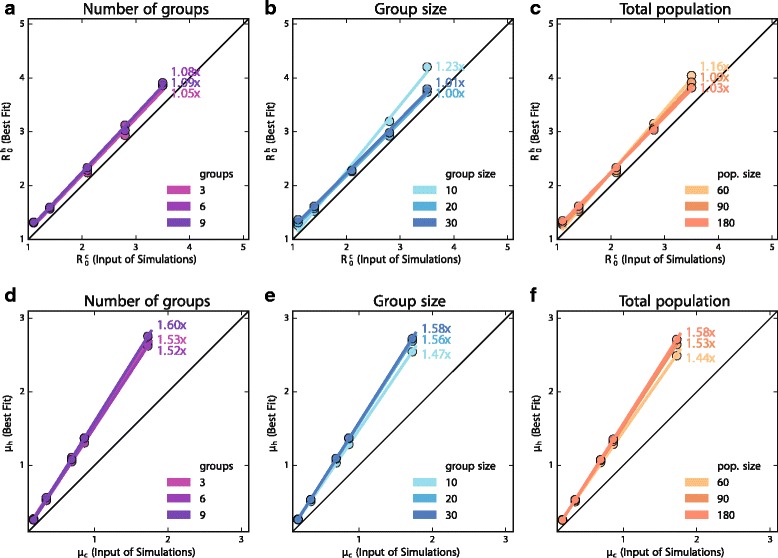
Recalibration of epidemic parameters for synthetic networks. **a**-**b**-**c**: Best fit values of the reproductive number ${R_{0}^{\mathrm {h}}}$ as functions of the corresponding input values ${R_{0}^{\mathrm {c}}}$. **d**-**e**-**f**: Best fit values of the recovery rate *μ*_*h*_ as functions of the corresponding input values *μ*_*c*_. Different population properties are explored: number of groups (**a**, **d**), group size (**b**, **e**), total population size (**c**, **f**). In each panel, each color corresponds to a specific value of the population property considered. Lines are the result of linear fits to the points obtained in each location using unweighted least squares

The linear dependency between the fitted parameters and the input ones is also found when synthetic models are used to define the contact patterns (Fig. 6). Moreover, the relations are quantitatively similar across groups, group sizes, and total population sizes, for both parameters. The average rescaling factor for the reproductive number is 1.06 (average value over all cases reported in Table 5, except the one with group size equal to 10, where the small group size might give rise to more important size effects), while for the average recovery rate it is 1.54. We also note that the scaling factors for the recovery rate in the synthetic cases are quite close to the one obtained in the conference setting.

**Table 5:** Values and standard deviations of the parameters *a*, *b*, *d*, *e* of the linear relations ${R_{0}^{\mathrm {h}}}=a\cdot {R_{0}^{\mathrm {c}}} + b$ and *μ*_h_=*d*·*μ*_c_+*e* obtained from the fits of Fig. 6

Groups	*a*	*b*	*d*	*e*
3	1.05±0.05	0.10±0.11	1.54±0.02	0.02±0.02
6	1.08±0.02	0.08±0.06	1.521±0.003	−0.004±0.002
9	1.07±0.03	0.106±0.07	1.60±0.01	−0.001±0.011
Group size				
10	1.23±0.06	−0.19±0.14	1.470±0.007	0.010±0.006
20	1.00±0.03	0.18±0.06	1.557±0.009	0.005±0.008
30	1.01±0.03	0.21±0.07	1.583±0.008	−0.005±0.008
Total size *N*				
60	1.16±0.05	−0.09±0.11	1.44±0.02	0.03±0.02
90	1.09±0.03	0.05±0.08	1.531±0.006	0.002±0.006
180	1.03±0.02	0.19±0.04	1.578±0.003	−0.012±0.003

## Discussion

The large availability of datasets exposing the contacts between individuals and the increasing computational ability have made individual-based models a powerful and widespread approach to describe infectious disease dynamics in a host population. This is particularly useful for simulating epidemics in closed settings, where detailed contact data may become accessible, sometimes including temporal resolution. The approach becomes however prohibitive when dealing with large-scale populations or multiple settings, as explicit data is generally missing. This is the case, for example, of spatially explicit agent-based models for the spread of respiratory infections in a given country [6–16]. In absence of data, homogeneous mixing models are then used. While they are known to provide important and valuable results for several epidemic aspects [1, 2], their application is challenged by their lack of realism in reproducing the mixing of individuals. While several previous works have addressed the issue of adjusting homogeneous mixing models to account for a non-trivial static structure of the contact network [17, 28, 29, 32], the recent development of the temporal networks field has shown that spreading processes on time-resolved data possess highly non-trivial properties [20, 33–39, 41, 42]. Here, we have therefore considered whether homogeneous mixing approximations can effectively reproduce epidemic processes unfolding on temporal networks. Through the study of empirical and synthetic temporal networks of contacts between individuals, we have shown that there exists an adjustment of the homogeneous mixing model meant to increase the model’s realism for describing epidemics unfolding on temporal networks of contacts in closed settings. The adjustment is based on the recalibration of the reproductive number and the recovery rate of the homogeneous mixing model to capture the dynamics simulated on the time-evolving contact patterns.

We found that fitting the average epidemic prevalence profile allows us to reproduce an average epidemic curve that has similar characteristics to the one unfolding on the contact network, even when the explicit details of the contact structure (i.e. its topology and its time evolution) are neglected. Higher accuracy is obtained in the prediction of the peak values compared to the peak times, the latter also depending on the context under study. This indicates that the recalibrated homogeneous mixing model approximates well the peak prevalence in the population in all investigated settings, while the time at which the peak is reached is more sensitive to the specific setting. Accuracy in capturing the epidemic size is harder to achieve with respect to the other two indicators, as expected given that the fitting was performed on the evolution of the density of infectious individuals and not on the density of recovered individuals. We found however that a regular relation exists between the epidemic size obtained from the recalibrated homogeneous mixing model and the one from the contact network model, thus providing an empirical law to estimate the realistic epidemic size from simpler simulations performed with a homogeneous mixing approach.

The accuracy of the homogeneous approximation is found to be fairly good also in the cases explored with the synthetic network model. Moreover, results are generally less variable, except for the accuracy in reproducing the peak time that is found to improve for larger populations (e.g. by increasing the group size or the number of groups). Larger discrepancies are found for the epidemic sizes, similarly to the findings obtained considering the empirical datasets, and a scaling relation exists between the two quantities also in the synthetic case. The relation appears to be almost linear in this case, compared to the slightly sublinear behavior found with the empirical datasets. This difference in behavior may be due to the simplified dynamics produced by the synthetic contact network model with respect to the realistic ones.

We found an intrinsic dependence among the variations, and between the variations and the reproductive number characterizing the epidemic under study. In general, the accuracy on the peak time increases at the expense of the accuracy in capturing the epidemic size and the peak prevalence. The error in the prediction of the epidemic size decreases for increasing reproductive number, showing a stronger robustness once more severe epidemics are considered. This is likely due to the fact that these epidemics are more stable in terms of epidemic indicators integrating the full dynamics, like the epidemic size. On the other hand, for a point value such as the peak value we find worse approximations. The observed behavior results from the quantity we considered to fit - the prevalence curve - and different behaviors of discrepancies may be expected in other fitting procedures. Note that, despite these dependencies, the fitting procedure cannot be optimized to reduce the discrepancy on a given epidemic feature. Our procedure indeed chooses the best overall fit to the whole prevalence curve, as we aim to reproduce the whole epidemic dynamics. Variations of the fitting procedure should be considered for alternative objectives.

Overall, our findings indicate that it is possible to recalibrate a homogeneous mixing model to reproduce a set of important features of the epidemic spread with median discrepancies <20 *%*, for all features, all datasets (empirical and synthetic) and all parameter values investigated.

Given these levels of accuracy, we assessed the features of the recalibrated homogeneous mixing model. Surprisingly, we found that a linear relation exists between the recalibrated disease parameters and the real ones, for both the reproductive number and the recovery rate. This means that, given an epidemic unfolding in a host population characterized by time-evolving contacts, it is possible to accurately describe that epidemic by means of a much simpler approach, the homogeneous mixing, once its parameters are rescaled according to such law. The great advantage offered by this linear relation is that it succeeds in capturing the disease dynamics across all probabilities of transmission and recovery rates explored. We found, however, that it depends on the empirical setting considered, suggesting that the recalibration of the homogenous mixing model is context-specific. This is expected given the different interaction dynamics occurring between individuals in the different settings, which makes for instance a school epidemiologically different from a workplace or a conference.

The epidemic appears to evolve faster in the temporal contact network than in a corresponding homogeneous mixing model with the same epidemic parameters. Though the unadjusted homogeneous approach assumes random mixing in the population, the fast dynamics of the real contacts established by individuals appear to be more efficient in the spread of the disease, reaching higher prevalences in a shorter time. We showed that this behavior can be accurately reproduced by the recalibrated homogeneous mixing. Interestingly, and different from previous results on static networks [17], we found that the tuning of both epidemic parameters is needed. An increase of the reproductive number is required to accurately describe the epidemic in the workplace and conference settings, whereas the opposite behavior is observed in the school. Adjustments of the recovery rate are also needed in the recalibrated homogeneous mixing model to adapt to the faster disease dynamics, even with a lower transmissibility. Shorter infectious periods are found to be always required by the recalibration across all settings, thus providing a higher turnover of infected into recovered that results in a shorter tail of the epidemic. Our results show how different outcomes may be achieved once epidemics spread on real contact networks, similar to previous work [34, 39]. In addition, they provide quantitative relations where such differences are reconciled and absorbed in the tuning of two epidemic parameters. The recalibration of only one of the parameters would not suffice to fit both the initial exponential growth and the tail of the epidemic as we are attempting to do here.

The recalibration is found to be more robust across the populations types considered in the synthetic networks. The rescaling coefficient of the reproductive number is slightly larger than 1 in all cases (with the exception of the group size of 10 individuals where a higher value may be induced by the small population size). This finding is likely due to the self-similar dynamics of interaction between individuals implemented in the synthetic contact networks. The dynamics is maintained the same across different groups, group sizes or total population sizes, so that similar quantitative results are expected once these indicators are changed. Small variations may simply be due to size effects.

The comparison of synthetic vs. empirical findings provide also insights into the mechanisms behind the resulting recalibrations. The fact that the scaling factors for the adjustment of the reproductive number are close to one in the synthetic cases, compared to stronger super linear behaviors observed in the workplace and conference settings, shows that a smaller adjustment of the reproductive number is needed once contacts are characterized by a simpler dynamics. Once non-random peculiar structures are considered, as those inevitably present in the empirical datasets, stronger adjustments in the transmissibility are required. The School dataset is the only case where an underestimation of the transmissibility is needed to reproduce the epidemic process, in contrast to all other cases explored. The study of additional datasets of contacts between students at various schools would be needed to understand whether the observed behavior is specific of this setting or of the particular dataset under study. The value of the rescaling factor is however very close to one, similarly to the values obtained in the synthetic cases (though smaller vs. larger than one). Synthetic contact networks share similar features with the contact patterns recorded at the school: first, the synthetic networks are composed of groups of the same size, similarly to classes at school but differently from the workplace departments, which have varying sizes; second, the synthetic contact matrix describing the interactions between and within groups (with only two values for *a*_*p,q*_) is more similar to the one observed in schools [47–49], than to the more heterogeneous and more mixed patterns observed at the workplace [50]. Schools composed of rather homogeneous substructures in terms of class sizes may thus be nicely described through our rescaling, regardless of the specific number of classes and class size. This is particularly relevant as schools represent an important mixing group for the transmission of airborne infections, due to a pattern of contacts that is strongly age-dependent [67–69]. Workplace results are different from school results, indicating that the same homogeneous approximation cannot be used in both settings (as currently done in the realm of agent-based models) to achieve accurate epidemic curves, but that a stronger rescaling of the basic reproductive number should be considered. We note however that repeated observations of empirical networks for each setting would be needed to assess the robustness of the recovered behaviour.

The infectious period is instead subject to a similar recalibration procedure for different types of contact networks. The slightly larger fluctuations observed for the case of empirical networks reflect stronger variations in network temporal patterns and timescales. Overall robustness and similarity are however found across all experiments, indicating that the specific dynamics of contacts becomes more relevant for the recalibration of the reproductive number.

Our study has a set of limitations that we acknowledge here. It does not consider the key mixing group represented by households. Contact data in households have up to now only been obtained through surveys, thus offering a lower temporal resolution with respect to the sensors used in the data collection experiments considered here. Few studies have focused on the extraction of an accurate representation of the contacts within a household for epidemic purposes [70, 71]. We argue that our approach may in any case not be ideal in this specific type of mixing context, given the small size of the household populations to be considered (see e.g., our results obtained with groups of 10 individuals).

The study considers face-to-face interactions between individuals as the only relevant contact for possible transmission of airborne infections. It is known however that other means of transmission may be relevant. In the case of influenza, for example, transmission may also occur through aerosol, i.e. small particles that remain airborne and can be carried over longer distances, and through indirect contact, i.e. passive transfer of viruses from an infected person to an uninfected individual through a contaminated surface. To model aerosol transmission, co-location of individuals may be used as a proxy for long-range contact for transmission. This information may be extracted in the datasets under study, however the model should also be informed with the relative force of transmission along this route. Transmission via contaminated surfaces would be harder to capture in terms of data. Few attempts in similar directions are now using sensors to detect proximity or contacts to specific objects (e.g. hand hygiene devices [72]), however the extension to all possible surfaces would require a radically different technological framework. It is important to note that few modelling studies have recently addressed the importance of multiple routes of influenza transmission [73]. Evidence suggests that all routes may be relevant, but their relative importance is highly dependent on parameters that are rarely available, or for which we have poor estimates, due to the difficulty of estimation from field data [73], so that no consensus on the issue has been achieved yet [74]. For this reason, we restricted the transmission of a generic airborne infection to the droplet transmission mechanism only, occurring through close proximity contacts, provided by the available data. We argue however that this does not represent a strong limitation of the study. Considering additional routes would indeed translate into integrating additional links to the network of contacts. While this would certainly alter the epidemic spread, here we consider this epidemic in a comparative way, identifying what adjustment is required for the homogeneous mixing model to capture the epidemic dynamics on the contact network. Therefore, we expect that changes to the contact network would be absorbed by the recalibration procedure. This is indeed confirmed by the successful performance of our procedure once different networks (with different densities and activities) are considered.

As previously mentioned, additional experiments on other contact datasets for these types of settings would be needed to further generalize our findings. The full length of the data collection may also be important. We found indeed in previous work that short timelines may alter some important aspects of the epidemic outcome (e.g. less than one day in a School example) [41]. For this reason, here we have considered the total time length for each dataset available, in order to be able to capture the full dynamics of the interactions among individuals, that shorter periods may hide. Only the Conference dataset has a shorter duration, of 1 day. We argue however that we expect to have a smaller bias in this setting, given that most of the novel interactions are established in the first day of the conference [20]. Longer datasets would be needed to extensively address this point.

## Conclusions

Our findings confirm that the homogeneous mixing model is only an approximation for the spread of an infectious disease in real locations of small and middle sizes. However, going beyond previous studies, our work highlights how a linear parameter rescaling can yield a good agreement between the epidemic curves obtained on a real contact network and with a homogeneous mixing approach in a population of the same size. This hints at the possibility of building a procedure based on the setting-specific linear rescaling of the reproductive number and of the recovery rate of an epidemic simulated on a given contact network in order to provide an accurate description of the time-evolution of the epidemic using the homogeneous mixing approach. However, to make this procedure more reliable, more information would be needed on contact patterns in the same settings to assess the robustness of the rescaling factors recovered here, in varying the specific workplace, school, etc, or a specific moment in time. We find low variability across different contexts in the values of the rescaling factors of the recovery rate, with respect to the ones of the reproductive number, probably since we are targeting social dynamics characterized by similar timescales of activity patterns. Larger, setting-specific variations of the rescaling factor for *R*_0_ are obtained, signaling the underlying differences in the contact dynamics across social contexts, and providing targeted solutions to simplify simulation modeling while preserving accuracy. These findings are critical for large-scale data-driven epidemic models, where specific contact data in different settings are missing. The use of the recalibrated homogeneous mixing approximation presented here may enhance the accuracy and realism of the simulations and limit the intrinsic biases of the homogeneous mixing.

## Additional file

Additional file 1 Supplementary Information containing additional Tables and Figures on correlation between relative variations *Δ* and epidemic parameters. (PDF 416 kb)
