# Supplementary material for: Recalibrating disease parameters for increasing realism in modeling epidemics in closed settings
Source: BMC Infect Dis. 2016 Nov 14;16:676. doi: 10.1186/s12879-016-2003-3 (PMC5109722; doi:10.1186/s12879-016-2003-3)
Supplement: Additional file 1 — Supplementary Information containing additional Tables and Figures on correlation between relative variations Δ and epidemic parameters. (PDF 416 kb) [file 12879_2016_2003_MOESM1_ESM.pdf]

Supplementary Information
Recalibrating disease parameters for increasing realism in modeling epidemics in closed settings

Livio Bioglio[1], Mathieu Génois[2], Christian L. Vestergaard[2], Chiara Poletto[3,4], Alain Barrat[2,5], and Vittoria Colizza[3,4,5]

[1]INVS: French Institute for Public Health Surveillance
[2]Aix Marseille Univ, Université Toulon, CNRS, CPT
[3]Sorbonne Universités, UPMC Univ Paris 06, UMR-S 1136, Institut Pierre Louis d'Epidémiologie et de Santé Publique, F-75013
[4]INSERM, UMR-S 1136, Institut Pierre Louis d'Epidémiologie et de Santé Publique, F-75013
[5]ISI Foundation

September 22, 2016

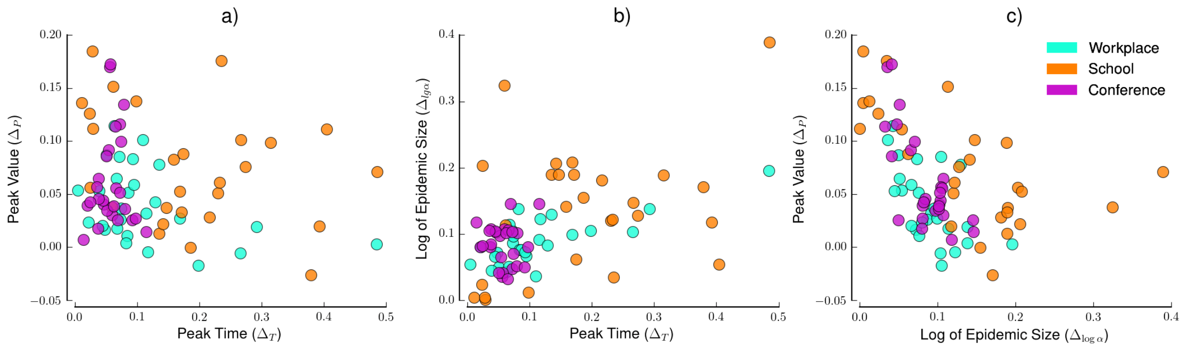

Figure S1: **Correlation between relative differences Δ.**

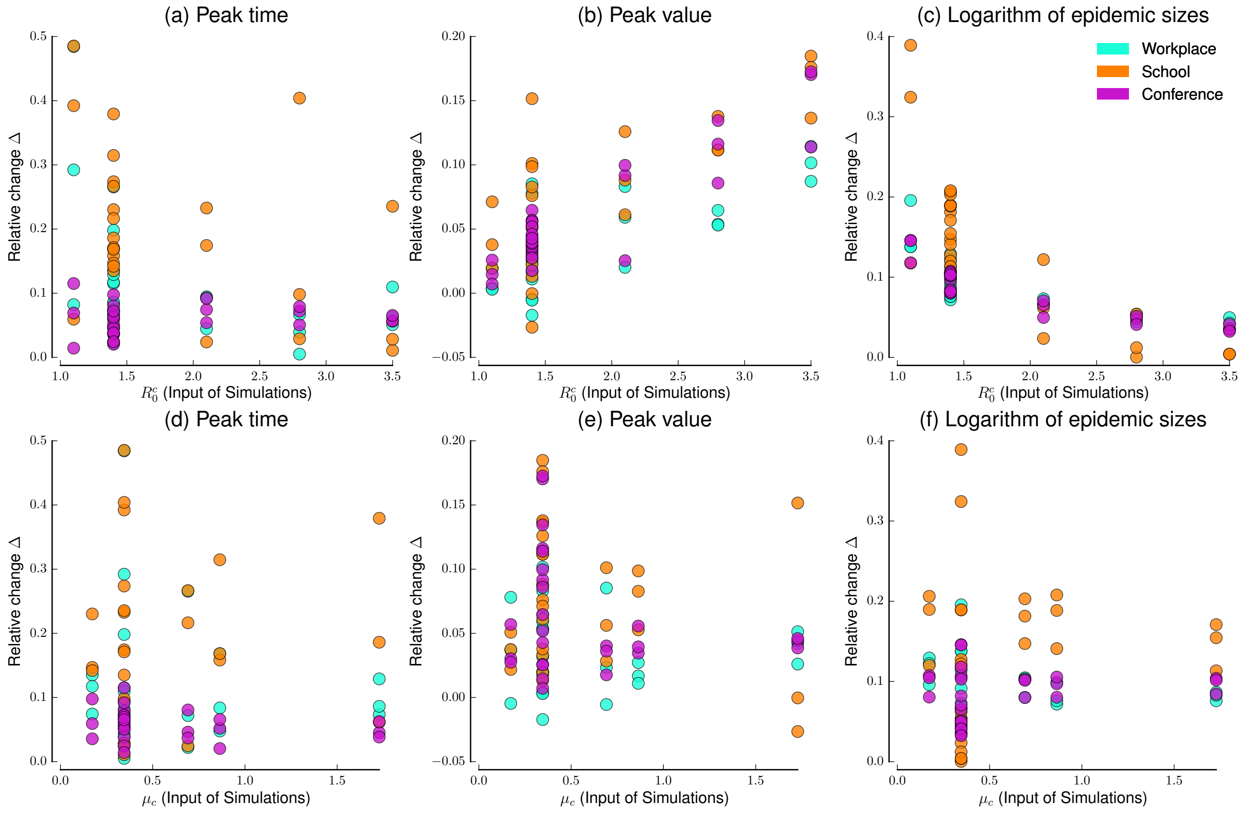

Figure S2: **Dependence of relative variations Δ on epidemic parameters.**

Table S1: **Correlations between relative variations Δ and reproductive number R_0^c.**

**Location (Full)**	Δ_T **vs.** R_0^c		Δ_P **vs.** R_0^c		$\Delta_{\log\alpha}$ **vs.** R_0^c	
	r[95%CI]	p-value	r[95%CI]	p-value	r[95%CI]	p-value
Workplace	$-0.6[-0.9, 0.2]$	0.1	$0.4[-0.3, 0.9]$	0.2	$-0.8[-1.0, -0.2]$	0.01
School	$-0.7[-0.9, -0.2]$	0.02	$0.6[-0.2, 0.9]$	0.1	$-0.9[-1.0, -0.6]$	$< 10^{-3}$
Conference	$-0.07[-0.70, 0.62]$	0.85	$1.0[0.9, 1.0]$	$< 10^{-3}$	$-0.9[-1.0, -0.7]$	$< 10^{-3}$

Table S2: **Correlations between relative variations Δ and recovery rate μ_c.**

**Location (Full)**	Δ_T **vs.** μ_c		Δ_P **vs.** μ_c		$\Delta_{\log\alpha}$ **vs.** μ_c	
	r[95%CI]	p-value	r[95%CI]	p-value	r[95%CI]	p-value
Workplace	$-0.2[-0.8, 0.5]$	0.6	$-0.3[-0.8, 0.4]$	0.4	$-0.1[-0.7, 0.6]$	0.8
School	$-0.2[-0.8, 0.5]$	0.6	$0.5[-0.2, 0.9]$	0.2	$0.3[-0.4, 0.8]$	0.4
Conference	$-0.3[-0.8, 0.4]$	0.4	$-0.3[-0.8, 0.5]$	0.5	$0.2[-0.6, 0.8]$	0.6
